# A Knowledge Management System for health emergencies: facilitating knowledge continuity and timely decision-making for frontline responders using experiential knowledge captured during action reviews

**DOI:** 10.3389/fpubh.2024.1427223

**Published:** 2024-08-27

**Authors:** Landry Ndriko Mayigane, Barbara Burmen, Armand Mbanya, Elliot Brennan, Candice Vente, Liviu Vedrasco, Stella Chungong

**Affiliations:** World Health Organization, Geneva, Switzerland

**Keywords:** “nuggets” of knowledge, learning, knowledge losses, knowledge failures, knowledge continuity, public health emergencies, health security, International Health Regulations (IHR) 2005

## 1 Introduction

### 1.1 Knowledge failures during past public health emergencies

The COVID-19 global pandemic has further exposed a volatile, uncertain, complex, and ambiguous world (1). The pandemic heightened the importance of accessing, processing, and disseminating available critical knowledge to guide emergency response actions to events in dynamic and uncertain times. At the center of the COVID-19 pandemic crisis has been the crisis of knowledge failure which countries have been maneuvering to remedy (2). Knowledge failures are not unique to the COVID-19 pandemic; they have also been evident during responses to past public health emergencies including previous coronavirus epidemics [i.e., the 2003 coronavirus causing severe acute respiratory syndrome, SARS-CoV, the 2012 Middle East respiratory syndrome coronavirus (MERS-CoV)] (3) and the 2018 Ebola virus disease (EVD) outbreak in the Democratic Republic of Congo (DRC) (4).

### 1.2 Learning from past public health emergencies

Nonetheless, knowledge from past emergencies and epidemics facilitated a rapid response to the COVID-19 pandemic, especially in its early phases, in some countries. Countries adjusted their policies based on past crises, such as the EVD outbreak in West Africa, the cholera outbreak in Haiti, the MERS and SARS outbreaks, and the H1N1 pandemic (5). Countries used any available knowledge to adapt their responses to programmatic and operational considerations brought about by the COVID-19 pandemic. For example, the pandemic influenza preparedness and response plan, developed and implemented before the COVID-19 crisis, was decisive in early country-level responses (6).

Similarly, following a previous Ebola virus disease (EVD) epidemic in Guinea, the country relied on lessons learned and capacities developed during the previous outbreak in a subsequent EVD epidemic (7). In this article, we describe current learning practices from health emergencies, their shortfalls and propose a knowledge management system (KMS) to facilitate effective knowledge management (KM) and KM continuity for public health emergencies management.

## 2 Current knowledge management practices

### 2.1 Information management systems used in public health emergency management

Information management systems (IMS), which have been in use for several decades (8), are data collection platforms that generate information to “*support strategic decisions, monitor changes, prioritize action and allocate resources, manage programs, scale up or scale down operations, advocate and formulate concerns in relation to an emergency context*” (9). IMS can either facilitate or hamper health emergencies' management. During the COVID-19 pandemic, IT-based systems facilitated prediction, diagnosis, treatment, infection prevention and health services management without which the pandemic would be difficult to control (10). During the 2014–2016 West Africa EVD outbreak, the existing clinical case record form was too research-focused, aggregate outbreak data collection tools could not be used for individual patients and siloed and fragmented data systems could not integrate all IMS elements hampering EVD control efforts (11).

### 2.2 Learning tools used during public health emergencies: action reviews

The World Health Organization (WHO) supports Member States to conduct Action Reviews (AR) including early-action reviews (EARs) (12), intra-action reviews (IARs) (13), and after-action reviews (AARs) (14, 15) to learn from on-going or past public health events. An EAR is conducted soon after an outbreak's onset to find system bottlenecks and fix them to prevent escalation of the outbreak (12). An IAR is periodically conducted during a protracted emergency to review and revise where appropriate, response actions taken to control the on-going emergency (13). An AAR is a post-outbreak assessment of response actions to identify gaps and best practices associated with unfavorable and favorable health emergency outcomes, respectively, to inform future preparedness and response actions. An AAR, which usually includes all relevant stakeholders and potential funders to finance the implementation of high-impact easy to implement AAR recommendations, formulates practical recommendations that are integrated into national annual or multiyear strategies to improve preparedness for and responses to future public health emergencies (15, 16). During action review and tabletop exercises (DARTs) retrospectively review past actions during emergency response and prospectively analyze future scenarios of concern in a table top exercise that is informed by the retrospective review to assess readiness and resilience (17). There are documented reports of EARs informing emergency responses in Cambodia and South Sudan (18), IARs leading to actionable items that resulted in better emergency outcomes in the USA and Kenya (19, 20), AARs enhancing the performance of public health emergency preparedness systems in the USA and other settings (21, 22), and DART in Bangladesh identifying opportunities for further transdisciplinary expert collaboration in its one health approach to COVID-19 (23). A review of 46 studies on AARs that had only quantifiable impacts done by teams or individuals over a wide variety of settings showed that action reports/debriefs improved effectiveness of individual and team performance and potentially public health emergency preparedness systems performance over a control group by ~25% (21).

### 2.3 Failure to capture tacit knowledge gained from past emergencies

Most countries conduct an AAR following a public health event and emergency (15). Inconsistent and unstandardized reporting systems limit the systematization of information gathered from AAR reports (24). AAR reports during the 2009 H1N1 pandemic, among other events, did not contain reflective root cause analyses of public health emergencies (24). Similar observations have been made by Becerra-Fernandez et al., who state that “…*action reports may not cover every issue that needs to be dealt with during an emergency, as frequently unique and unanticipated events arise during each emergency. Furthermore, people may leave the organization, due to attrition or retirement, and some of the informal rules that serve as the “glue” that affords the very ability to function may be lost*” *(**25**)*.

The quest for documenting contextual and experiential knowledge from the response to emergencies was highlighted during the 2021 WHO-supported AAR of four EVD outbreaks in the Democratic Republic of Congo. Health officials present at the AAR expressed a desire to systematically collect knowledge from many of the 2,000 responders during the outbreaks between 2018 and 2020 who had already left the country and were not present at the AAR. One official succinctly reflected that: “*The knowledge they [responders] gained from the response would be valuable only if made easily accessible for the country to use and support national efforts to better prepare for and respond to future Ebola outbreaks and other emergencies, and to build overall capacities for emergency management in the country before it is 'lost' forever”* *(**26**)*. Such knowledge is termed tacit knowledge. Tacit knowledge includes mental models, perspectives, intuitions, know-how and experiences and is difficult to formalize or communicate. Conversely, explicit knowledge is conveyed in formal systematic formats that are easy to communicate such as databases, procedures etc. (27). Tacit knowledge, which is dependent on socialization and externalization (i.e., the number of meetings or discussions that could encourage knowledge flow), rapidly diminishes in situations (or organizations) that experience rapid changes in roles or positions related to a specific workflow process (28). The high turnover of responders during emergencies erodes institutional memory and limits countries' ability to maintain momentum in their response (29).

The lack of systems for the timely capture of knowledge, including knowledge gained through the lived experiences of responders, may lead to knowledge discontinuity and a vacuum where the knowledge is most needed, both within and between responses. However, knowledge continuity, together with the right personnel, can help an organization to rapidly adapt to external conditions beyond its control such as public health emergencies. Individual level factors like willingness to share knowledge influence knowledge continuity (30). While there are in-country efforts to facilitate knowledge continuity, global mechanisms for cross border knowledge sharing are yet to be established (15, 31). Should countries be viewed as an “organization” with the World Health Organization serving as a secretariat, and organizational factors such as organizational culture influencing knowledge continuity (32), the World Health Organization can support knowledge continuity via its knowledge culture (33–35). Although knowledge losses may occur when members of an organization are disconnected (28), the World Health Organization can use its convening power (32) to harness this experiential knowledge of emergency response personnel in globally accessible platforms.

## 3 A Knowledge Management System (KMS) to address knowledge discontinuity in health emergency preparedness

A knowledge management system facilitates organizational learning, retrieval and reuse of knowledge assets by instituting “*initiatives, processes, strategies, and systems that sustain and enhance the storage, assessment, sharing, refinement, and creation of knowledge*” (36). The World Health Organization is developing a KMS to facilitate knowledge continuity and timely decision-making using experiential knowledge emerging from action reviews (12–14). The proposed KMS will capture contextual tacit knowledge from front-line responders involved in a specific public health event and summarize them as “nuggets” or “digestible” content (small pieces of information). “Nuggets” of knowledge (NoKs) will be stored on a searchable platform powered by taxonomy and anthology, and other content management technologies to maximize its accessibility, retrievability, and reusability in other contexts to inform emergency responses. The KMS will complement existing incident command and management systems (37) or any response or knowledge management systems (38) being used by countries.

The KMS will have the following essential components listed below (39):

- People: the KMS secretariat (and other stakeholders) will work with all emergency responders who have been involved responding to past public health events who will be contributors and users of the knowledge harnessed. The KM platform will be initially managed by a WHO secretariat with plans to expand its management to multiple stakeholders who will also act as administrators with WHO retaining its secretariat role.
- Processes: Knowledge curation, codification and diffusion activities will be conducted to obtain NoKs. Moreover, process monitoring and evaluation activities will help measure knowledge flows.
- Content/technological resources: the NoK platform will be an open-source digital platform that will avail the right knowledge at the right time to emergency responders to support planning, decision-making and knowledge continuity. The platform will be an abridged version of lessons learned from past and protracted health emergencies. The NoK platform will facilitate learning from EAR reports (12, 40), IAR reports (13), and AAR reports (14, 15) among other knowledge resources. Knowledge captured will be garnered as “digestible” content (NoKs) within an accessible collaborative and interactive platform for countries and key responders.

- NoKs generation will be tied to the World Health Organization's Disease Outbreak News (WHO DON) where WHO publicizes information public on acute public health events or potential events of concern (41). For non-infectious disease events, the activation of the Early Warning, Alert and Response Systems (EWARs) will trigger the plan to capture and generate some NoKs (42). Member States are advised (not required) to report all Action Reviews conducted under the IHR (2005) (43). Therefore, the plan to conduct an Action Review (EAR/IAR and AAR) will trigger the timely development of NoKs.
- The WHO secretariat will either receive contributions from emergency responders working at operational or policy level, or interview subject matter experts to tap into their memories of past events, or invite voluntary contributions, or coordinate activities to generate NoKs like knowledge jams, or generate NoKs from action review reports and published literature. All submitted NoKs will be validated by reviewers who are in-country subject matter experts and emergency response personnel to address authenticity and liability concerns, respectively, prior to publication. All published NoKs will be reviewed on a regular basis prior to retiring them from the platform to accommodate for the volatile nature of knowledge if necessary.
- The NoK platform will incorporate text, audio and visual NoKs which will all be open to the public who can anonymously access the client-facing platform to read published NoKs. However, users would need to register to submit NoKs. Since this is an open-source platform, data will belong to Member States who can access it as often as needed.

- Knowledge management culture: the World Health Organization echoes the value of knowledge in attaining its mission (33–35) by supporting countries to conduct EARs (12, 40), IARs (44), and AARs (14) among other activities.
- Strategic vision: the strategic vision of the KMS that includes the NoK Platform aligns with the World Health Organization's vision, mission, objectives, strategy and approach in developing a KMS for public health emergency preparedness.

**Figure 1:**
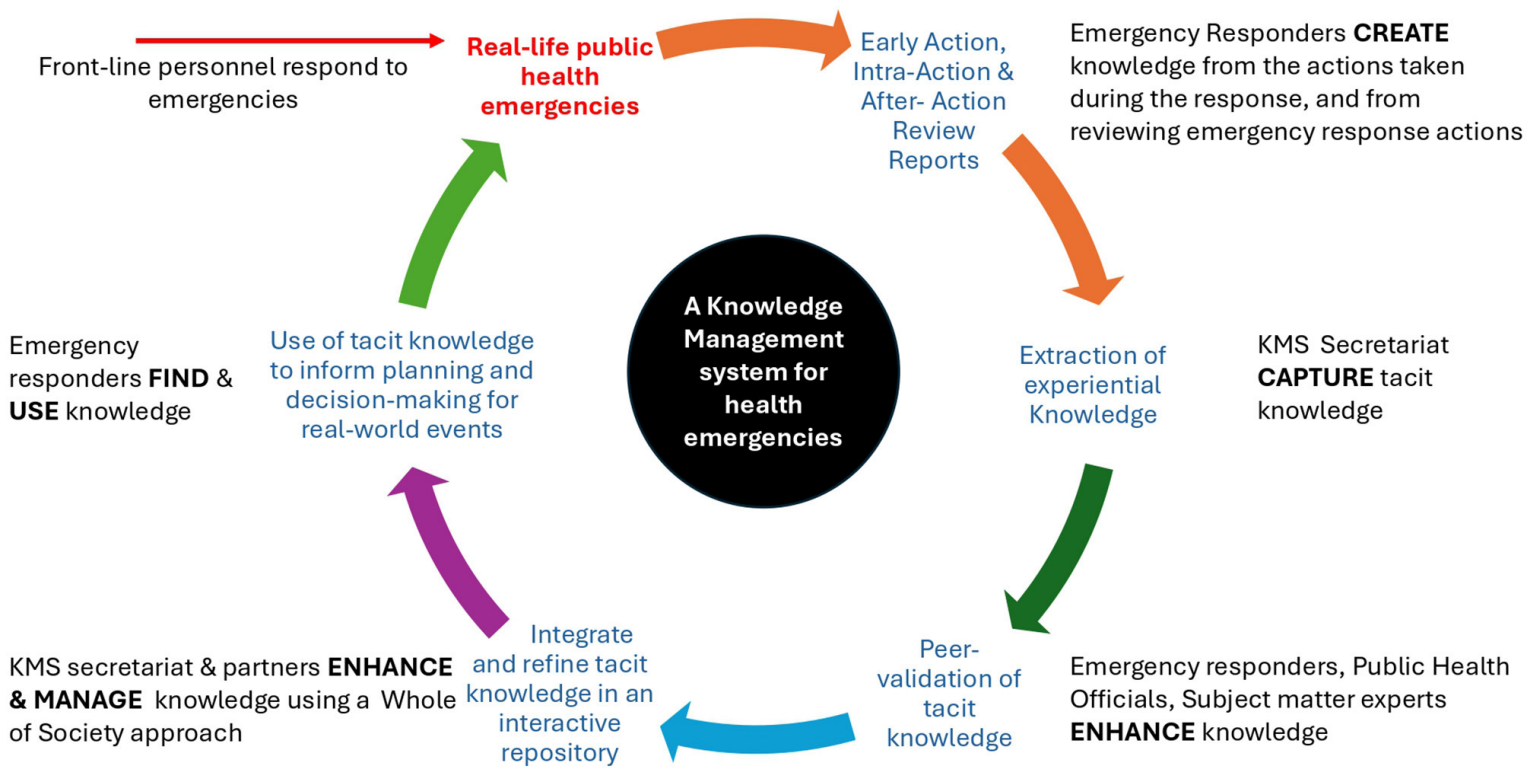
A knowledge management action wheel for the NoK Platform for health emergencies. The WHO echoes the value of knowledge in attaining its mission by supporting countries to conduct action reviews to learn from responses to real-life emergencies. Walh (45) propose a knowledge action wheel to ensure KMS enable specific actions and results. The proposed KMS, will build on routine learning processes to facilitate learning the reuse of experiential knowledge from health emergencies based on processes in the KM action wheel proposed by Walh (45). Adapted from: Walh (45).

The proposed KMS, illustrated in the KM Action wheel (45) shown in Figure 1, encompasses the creation of NoKs, the capturing of NoKs on the platform, the validation and enhancement of NoKs, the management of Noks on the platform, and the retrieval and reuse of NoKs.

## 4 Discussion

### 4.1 A Knowledge Management System as a critical incident registry for public health emergency preparedness

**Figure 2:**
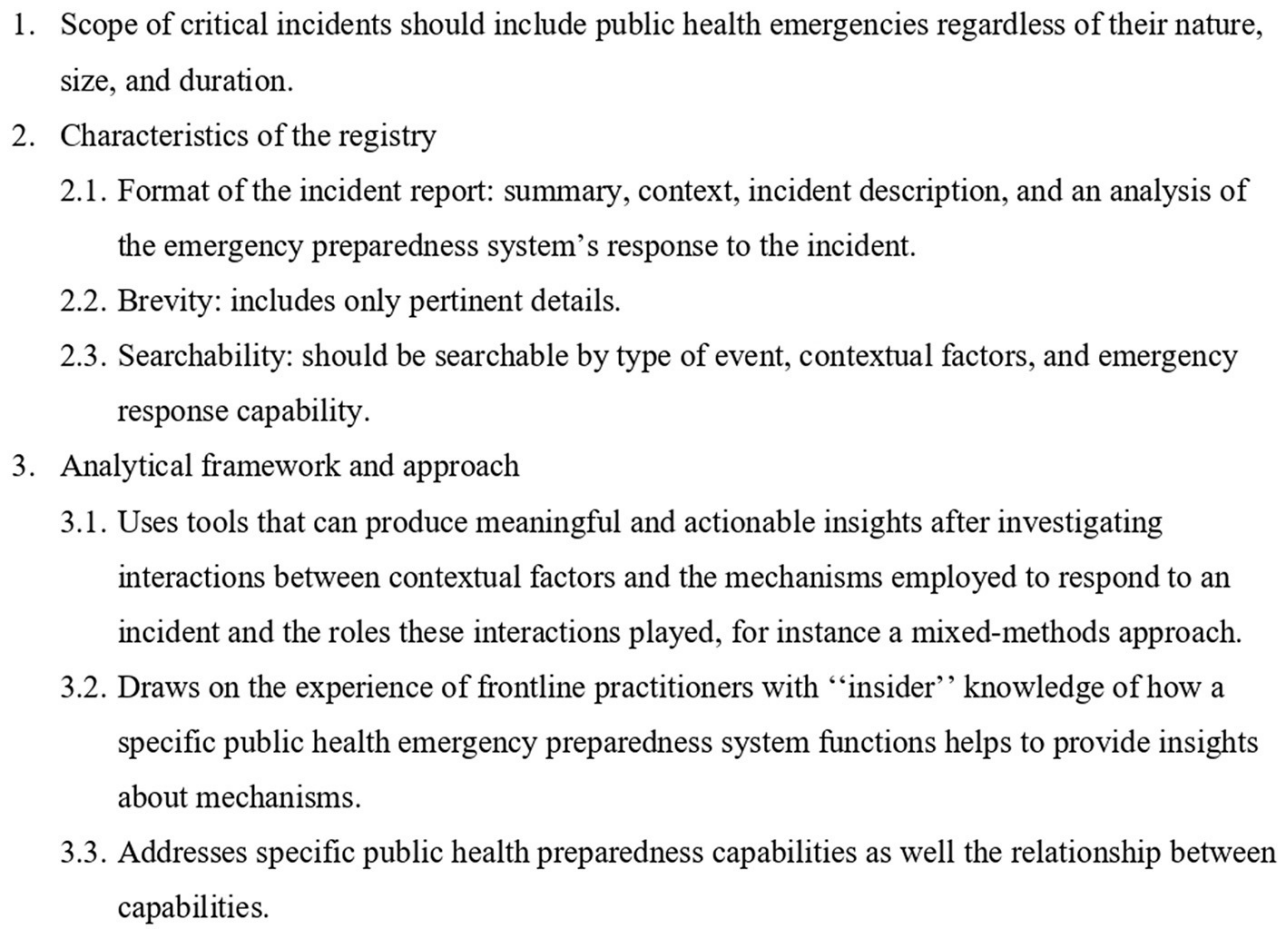
Elements of a critical incident registry. Piltch-Loeb et al. (24) propose the use of a Critical Incident Registry for Public Health Emergency Preparedness, that has been used in the aviation industry, to address the ongoing knowledge loss in public health emergency management. The proposed KM aims to allow countries to access and turn knowledge from past public health events into assets that they can harness at any time. The proposed KMS will employ elements of a critical incident registry proposed by Piltch-Loeb et al. (24) to address existing knowledge losses in public health emergency management in a country from one outbreak to another. Adapted from Piltch-Loeb et al. (24).

Piltch-Loeb et al. (24) proposed the use of a Critical Incident Registry for Public Health Emergency Preparedness to address the ongoing knowledge losses in public health emergency management. Critical incident registries could help facilitate learning from public health emergencies by disseminating lessons learned from previous (and possibly ongoing) public health emergencies and translating these lessons to new incidents or new settings (24). The proposed KMS for health emergencies will build on the concept of critical incident registries proposed by Piltch-Loeb et al. (24) (Figure 2) to address existing knowledge losses in public health emergency management in a country from one outbreak to another.

The KMS will focus on public health emergencies which the World Health Organization defines as situations that have an immense impact on the health and lives of many people which require extensive intervention by multiple sectors (46) (**Element 1**). The KMS will employ a predominantly qualitative approach to probe how and why things happened, including knowledge “jams,” key informant interviews and action reviews (EARs, IARs, and AARs) (47) (**Element 2.1** and **3.1**). The KMS will harness NoKs from action review reports that have been prepared by health emergency front-line responders who were involved in a specific emergency response (**Element 3.2**) and structure them “digestible” content (“nuggets”, i.e., small pieces of information; **Element 2.1 and 2.2**) in a searchable platform using various categories (**Element 2.3**). The “nuggets” will be categorized based on IHR core capacities and technical areas in the recently published WHO Benchmarks for strengthening health emergency capacities (31) (and other taxonomy, including the emerging pillars highlighted in the COVID-19 pandemic pillars as key priorities by the WHO to control COVID-19, a.k.a., the COVID-19 pillars (48), as well as taxonomy covering the time, space, and persons/populations affected by an emergency) which are “meaningful” for the identification, evaluation, and notification of events and for responding to public health risks and emergencies (**Element 3.1**) (49). The categorization of NoKs will facilitate the identification, retrieval, evaluation, and linkages amongst events to provide insights for responding to public health risks and emergencies based on life-saving decisions taken in different contexts. Information will be listed on the NoK platform only after rigorous scrutiny and validation to ensure that it is specific enough (in terms of people's mix, e.g., multisectoral and multidisciplinary collaborations; purposes, e.g., repurposed or downcycled; and processes, e.g., unconventional, or innovative processes, places, or contexts), to elicit specific responses **(Element 3.3**).

### 4.2 Prospects of the Knowledge Management System

The evidence-based KMS will serve a learning hub for public health practitioners, policymakers, and the broader community by providing “bite-size chunks” of information to limit the information overload experienced by emergency response personnel during crises when they need to make “*accurate decisions, under time-pressured and intense situations*” (50). The KMS will allow countries to access and turn knowledge from past public health events into assets that they can harness at any time.

Additionally, the Nok platform has significant potential to leverage technologies such as artificial intelligence (AI) in resolving knowledge discontinuity and maintaining “living” memory and knowledge in emergency management (51). AI can be used in different KM processes. AI can be used for predictive analytics and natural language processing when obtaining knowledge from different sources during knowledge creation, to structure knowledge using various ontologies and present knowledge in various formats during knowledge classification, organization, storage and retrieval, and to integrate siloed systems and permit real-time smart-sharing of knowledge and interactive feedback during knowledge sharing. Furthermore, AI could facilitate knowledge application by using context-tracking mechanisms to detail intermediate processes through which information moves from data mining to knowledge discovery to business rules with a view to avail situated (contextual) knowledge to the right person at the right time (52, 53). All data generated using automated algorithms will be moderated prior to dissemination on the NoK platform (54).

Given cultural, political and other different dynamics within countries and the potential reluctance in information sharing between countries, countries should first strive to develop in-country knowledge management and knowledge continuity practices to maximize in-country contextual learning (30). For instance, information collected in after action review reports could be gathered in a database of reports, to connect past experiences to future improvements (22). Secondly, countries could share permissible knowledge across countries to facilitate peer-to-peer learning (15, 31).

## 5 Conclusion

A growing body of literature acknowledges that learning health systems are robust health systems (55). Stoto et al. refer to AARs without learning as “box-checking” exercises (21). Therefore, as health systems recover from COVID-19 and other health emergencies, it is imperative that aggregated findings and lessons learned from EARs, IARs and AARs of COVID-19, or other public health events, are captured and used as the foundation for active learning practices to avoid the “panic-then-forget” cycle of emergency response (56). Such knowledge will prevent emergency responders from “re-inventing the wheel” during each subsequent emergency and support countries to build sustainable capabilities for emergency management. Ultimately, the proposed KMS platform seeks to have a far-reaching impact on the emergency management cycle by supporting knowledge continuity in countries for broader global health security.

## Author contributions

LM: Writing – review & editing, Conceptualization, Writing – original draft. BB: Visualization, Writing – review & editing. AM: Writing – review & editing. EB: Writing – review & editing. CV: Writing – review & editing. LV: Writing – review & editing. SC: Conceptualization, Writing – original draft, Writing – review & editing.
